# The Influence of Insulin Resistance and Type 2 Diabetes on Cognitive Decline and Dementia in Parkinson’s Disease: A Systematic Review

**DOI:** 10.3390/ijms26168078

**Published:** 2025-08-21

**Authors:** Osama Zeidan, Noor Jaragh, Maya Tama, Maryam Alkhalifa, Maryam Alqayem, Alexandra E. Butler

**Affiliations:** 1 School of Medicine, Royal College of Surgeons in Ireland, Medical University of Bahrain (RCSI-MUB), Adliya P.O. Box 15503, Bahrain; 21202365@rcsi-mub.com (O.Z.); 21201204@rcsi-mub.com (N.J.); 22200235@rcsi-mub.com (M.T.); 21202369@rcsi-mub.com (M.A.); 21200156@rcsi-mub.com (M.A.); 2 Research Department, Royal College of Surgeons in Ireland, Medical University of Bahrain (RCSI-MUB), Adliya P.O. Box 15503, Bahrain

**Keywords:** insulin resistance, type 2 diabetes, cognitive decline, dementia, Parkinson’s disease

## Abstract

Parkinson’s disease (PD) is a common neurodegenerative disorder caused by progressive loss of dopaminergic neurons in the substantia nigra and the presence of Lewy bodies. While PD is most recognized by its motor symptoms (resting tremor, rigidity, bradykinesia, and postural instability), cognitive decline (CD) may become apparent as PD progresses, leading to Parkinson’s disease dementia (PDD). Type 2 diabetes mellitus (T2DM) and insulin resistance (IR) are risk factors for dementia, especially Alzheimer’s disease; however, their influence on dementia in PD is underexplored. Therefore, we sought to determine the effect of T2DM and IR on dementia in PD. A systematic search of articles from 2005 to March 2025 was undertaken using Embase, PubMed, Scopus, Web of Science, and citation searching. Case–control, cross-sectional, longitudinal, and non-human population studies assessing cognitive outcomes in individuals with PD, with and without T2DM and IR, were included (PROSPERO registration number CRD420251013367). In total, 27 studies met the inclusion criteria, with clinical sample sizes ranging from 23 to 544,162 participants. Among the 23 clinical studies, 15 identified T2DM as a contributor to cognitive decline (CD) in PD, and 4 specifically examined insulin resistance (IR). Elevated HbA1c was consistently associated with poorer cognitive performance and increased risk of Parkinson’s disease dementia (PDD); HbA1c ≥ 7% independently predicted cognitive impairment (OR = 4.25, 95% CI: 1.59–11.34). Vascular and inflammatory markers, including elevated LDL-C, fibrinogen, and hs-CRP, further exacerbated CD. MoCA and MMSE scores were the most common cognitive measures, consistently showing worse outcomes in PD patients with T2DM. Preclinical studies supported these associations, showing that high-fat-diet-induced T2DM and IR aggravated dopaminergic neuronal loss by 38–45%, increased α-synuclein by 35%, and heightened microglial activation, providing mechanistic evidence for the observed clinical associations. This systematic review, the first to examine the impact of T2DM and IRs on the occurrence and advancement of CD in PD patients, demonstrates a possible association between the two. However, these results demonstrate the need for larger sample sizes and the inclusion of additional clinical variables, such as HbA1c levels and pharmacological interventions, providing further information about the link between metabolic dysfunction and CD in PD. To further strengthen this link, longitudinal studies with systematic follow-ups are essential to establish causal links and avoid misdiagnosis in clinical practice.

## 1. Introduction

Parkinson’s disease (PD) is a common neurodegenerative disorder characterized by the progressive loss of dopaminergic neurons in the substantia nigra and the presence of Lewy bodies. Parkinson’s disease is a synucleinopathy, marked by atypical aggregation of α-synuclein in a prion-like manner [1]. Clinically, it primarily manifests with motor symptoms such as bradykinesia, resting tremor, rigidity, and postural instability [2]. However, PD is not confined to motor impairment; its slow progression often leads to cognitive decline, ultimately resulting in Parkinson’s disease dementia (PDD) [3]. The degree of cognitive impairment in PD is highly variable, ranging from mild cognitive dysfunction to severe, debilitating dementia [4]. In recent years, growing attention has been directed toward understanding the cognitive aspects of PD, as these symptoms significantly affect patients’ quality of life and increase caregiver burden.

While most PD cases are idiopathic, Parkinsonian symptoms can also be induced by external factors such as certain medications. Regardless of cause, cognitive decline remains a serious and common complication of PD progression [5]. This introduces a crucial question: what factors may accelerate or contribute to this cognitive deterioration?

Type 2 diabetes mellitus (T2DM) and insulin resistance (IR) are two increasingly prevalent metabolic disorders. Insulin, a key peptide in glucose metabolism, has also been shown to influence central nervous system function, regulating cognitive processes and affecting both glial and neuronal metabolism [6]. IR, defined as reduced sensitivity to insulin, has repeatedly been proposed as a major risk factor for cognitive decline [7,8]. Similarly, T2DM has been associated with mild cognitive impairment and an increased risk of dementia in multiple studies [9]. While several mechanisms, such as chronic inflammation, oxidative stress, and vascular injury, have been proposed to explain this association, the effect of T2DM and IR on dementia specifically in PD remains underexplored [10,11].

Although one prior systematic review investigated general risk factors for cognitive impairment in PD, T2DM and IR were not the primary focus [12]. Therefore, this systematic review is necessary to determine whether T2DM and/or IR serve as significant risk factors in the development of cognitive decline and/or PDD in patients with PD.

By systematically synthesizing and critically evaluating the current evidence, this study aims to produce a comprehensive understanding of the relationship between T2DM, IR, and cognitive outcomes in Parkinson’s disease. Furthermore, it aims to explore potential underlying mechanisms linking these metabolic conditions to neurodegeneration in PD, offering insight to support clinical decision-making, therapeutic strategies, and future research.

## 2. Methods

### 2.1. Inclusion Criteria

Studies were selected based on specific PECO (population, exposure, comparison, and outcomes) criteria (Table 1). Eligibility criteria included cross-sectional, prospective, and retrospective studies and studies involving non-human populations. Only English-language studies were considered. We restricted our literature search to publications from the last 20 years because the Montreal Cognitive Assessment (MoCA) was introduced and validated in 2005 by Nasreddine et al. [13]. This cognitive screening instrument was widely adopted in the literature due to its greater sensitivity in detecting mild CD compared to MMSE in PD, especially in non-demented patients (32% vs. 11%, respectively) [14].

### 2.2. Search String

#### 2.2.1. MeSH

((“Diabetes Mellitus, Type 2”[Mesh] OR “Insulin Resistance”[Mesh] OR “Glucose Intolerance”[Mesh] OR “Diabetes Mellitus, Non–Insulin-Dependent”[Mesh]) OR (“diabetes type 2”[Title/Abstract] OR “type 2 diabetes”[Title/Abstract] OR “non-insulin dependent diabetes”[Title/Abstract] OR “adult onset diabetes”[Title/Abstract] OR T2DM[Title/Abstract] OR insulin[Title/Abstract] OR “insulin sensitivity”[Title/Abstract] OR “glucose intolerance”[Title/Abstract])) AND ((“Parkinson Disease”[Mesh] OR “Parkinsonian Disorders”[Mesh]) OR (“Parkinson’s disease”[Title/Abstract] OR “Parkinsonism”[Title/Abstract] OR “Parkinsonian syndrome”[Title/Abstract] OR “idiopathic Parkinson’s”[Title/Abstract] OR “Parkinson’s dementia”[Title/Abstract] OR “Parkinson’s cognitive impairment”[Title/Abstract] OR “Parkinson-related dementia”[Title/Abstract])) AND ((“Dementia”[Mesh] OR “Cognitive Dysfunction”[Mesh]) OR (“cognitive decline”[Title/Abstract] OR “dementia”[Title/Abstract] OR “mental deterioration”[Title/Abstract] OR “cognitive impairment”[Title/Abstract] OR “mild cognitive impairment”[Title/Abstract] OR “cognitive dysfunction”[Title/Abstract] OR “memory loss”[Title/Abstract] OR “neurocognitive decline”[Title/Abstract])).

#### 2.2.2. Non-MeSH

((“diabetes type 2”[Title/Abstract] OR “type 2 diabetes”[Title/Abstract] OR “non-insulin dependent diabetes”[Title/Abstract] OR “adult onset diabetes”[Title/Abstract] OR T2DM[Title/Abstract] OR insulin[Title/Abstract] OR “insulin resistance”[Title/Abstract] OR “insulin sensitivity”[Title/Abstract] OR “glucose intolerance”[Title/Abstract])) AND ((“Parkinson’s disease”[Title/Abstract] OR “Parkinsonism”[Title/Abstract] OR “Parkinsonian syndrome”[Title/Abstract] OR “idiopathic Parkinson’s”[Title/Abstract] OR “Parkinson’s dementia”[Title/Abstract] OR “Parkinson’s cognitive impairment”[Title/Abstract] OR “Parkinson-related dementia”[Title/Abstract])) AND ((“cognitive decline”[Title/Abstract] OR “dementia”[Title/Abstract] OR “mental deterioration”[Title/Abstract] OR “cognitive impairment”[Title/Abstract] OR “mild cognitive impairment”[Title/Abstract] OR “cognitive dysfunction”[Title/Abstract] OR “memory loss”[Title/Abstract] OR “neurocognitive decline”[Title/Abstract])).

A systematic review search was performed using the most recent and updated studies that have been published in the last ten years. Since there was only one systematic review examining multiple risk factors impacting cognitive loss in PD, and diabetes mellitus was one of them [12], this time frame was selected, and the focus was limited to DM and IR.

### 2.3. Study Selection

Study titles and abstracts were examined to ensure relevance. Conflicts regarding eligibility were resolved through discussion and resolution by two independent reviewers. Following initial screening, full-text articles were assessed using the pre-defined inclusion and exclusion criteria (Table 1).

### 2.4. Data Extraction

In total, 27 studies were included in this systematic review. Data extraction was performed using a standardized template designed to capture relevant study details. The extraction template included several key categories for data collection, such as study identification, population characteristics, exposures, and outcomes; strengths and limitations; and mechanisms of pathophysiology.

### 2.5. Quality Assessment

A quality assessment was conducted using the Quality in Prognostic Studies (QUIPS) tool. Risk of bias was assessed across six domains: study participation, study attrition, prognostic factor measurement, outcome measurement, study confounding, and statistical analysis/reporting. Each study was rated as “high,” “low,” or “unclear.” Discrepancies between reviewers were resolved through consensus discussions.

## 3. Results

**Figure 1:**
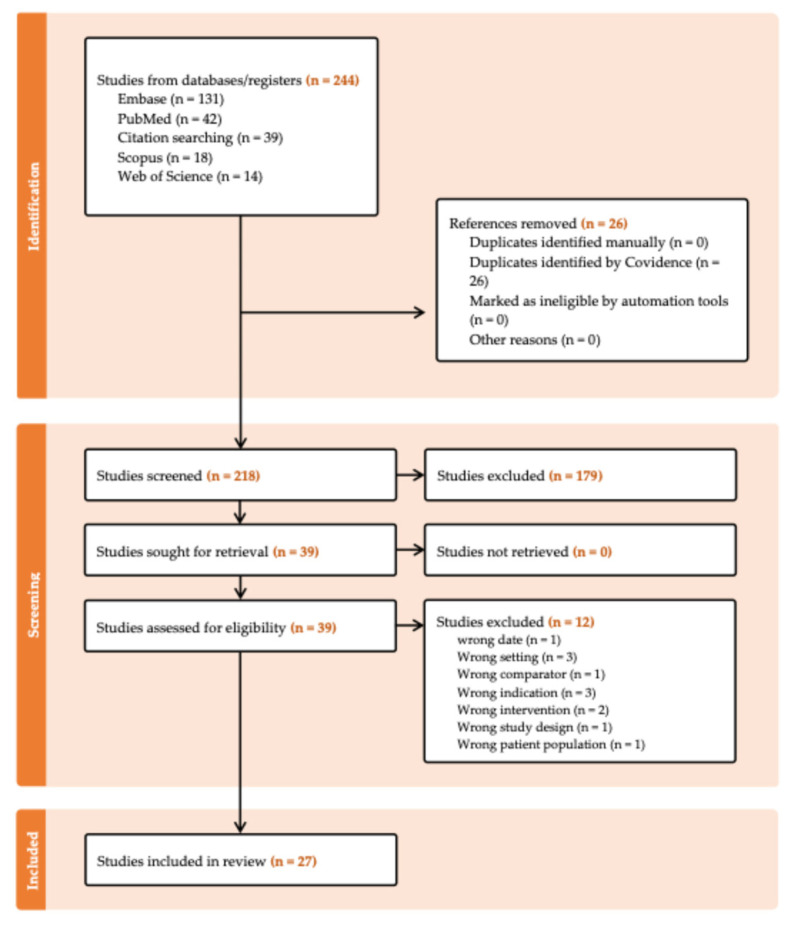
PRISMA flowchart.

Our search yielded 244 results, 26 of which were duplicates. Of the remaining 218 studies, 179 were excluded during abstract screening. In total, 39 studies underwent full-text screening, with 12 being excluded, yielding 27 studies for inclusion in this review. The study selection process is documented using the Preferred Reporting Items for Systematic Reviews and Meta-Analyses (PRISMA) flowchart, shown in Figure 1.

### 3.1. Characteristics of Selected Studies

Table 2 delineates the study characteristics. The studies included cross-sectional (*n* = 15), retrospective (*n* = 6), prospective (*n* = 2), and preclinical (*n* = 4) study designs. Sample sizes displayed variation: most included between 73 and 1930 participants, though some had significantly smaller or larger sample sizes. Two studies had very small sample sizes of 23–36 participants, the first due to recruitment from a single center over a relatively short period of 7 months [15], while the small sample size in the second can be attributed to the study design [16]. Two retrospective cohort studies had large sample sizes of between 147,096 and 544,162 participants with a relatively long follow-up period [17,18]. Most studies focused on older adults, the mean age of participants being 59–79 years.

**Figure 2:**
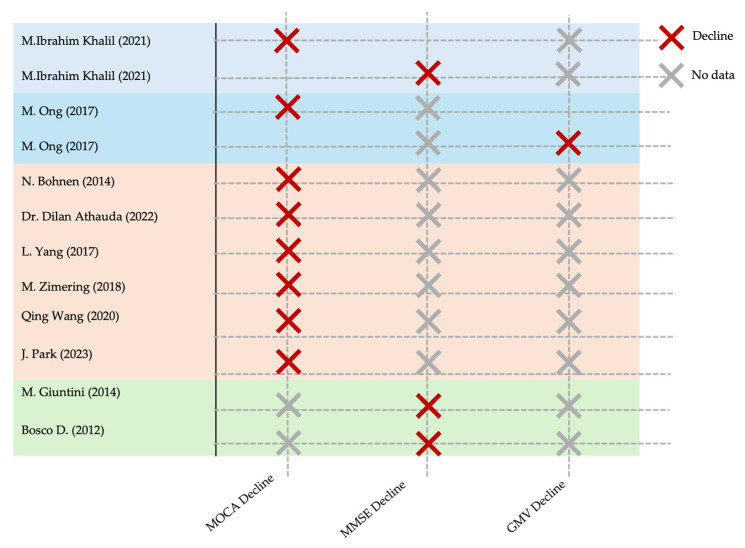
Summary of cognitive outcomes in clinical Studies of T2DM/IR in Parkinson’s disease. Abbreviations: MoCA = Montreal Cognitive Assessment; MMSE = Mini-Mental State Examination; GMV = gray matter volume. M. Ibrahim Khalil, 2021, Bangladesh [22]; M. Ong, 2017, Singapore [27]; N. Bohnen, 2014, USA [24]; D. Athauda, 2022, UK [31]; L. Yang, 2017, China [34]; M. Zimering, 2018, USA [15]; Q. Wang, 2020, China [32]; J. Park, 2023 [19]; M. Giuntini, 2014 [28]; D. Bosco, 2012, Italy [25].

A qualitative summary of each included study, encompassing participant characteristics, exposure definitions, measurement tools, and key cognitive outcomes, is presented in Table 3. To complement this, Figure 2 provides a semi-quantitative synthesis of the primary cognitive outcome measures and their reported direction of change. This format enables quick comparison across studies and facilitates identification of the most consistently reported cognitive domains affected by T2DM or IR in Parkinson’s disease.

Across the 27 clinical studies, the most frequently assessed cognitive domains were global cognition; the Montreal Cognitive Assessment (MoCA); the Mini-Mental State Examination (MMSE); and specific neuropsychological measures such as memory recall, attention, and executive function. Multiple studies demonstrated that PD patients with T2DM performed significantly worse on the MoCA; for example, J. Park et al.’s study reported a MoCA reduction of more than 2 points in T2DM and the prediabetes groups (*p* = 0.002) [19], while L. Yang et al.’s study found MoCA scores negatively correlated with both HbA1c levels and HOMA-IR [34]. In addition, Bosco et al. and Arthur Oscar Schelp et al. demonstrated that higher IR scores were strongly linked to lower MMSE scores and poorer memory performance [25,35].

Structural imaging findings were consistent with cognitive decline: M. Ong et al. reported significantly lower gray matter volume in T2DM-PD patients [27], and M. Petrou et al. observed greater gray matter loss in T2DM-PD compared with PD-only controls [16]. Similarly, Brit Mollenhauer et al. identified sleep-related biomarkers (elevated periodic limb movement index) associated with subsequent cognitive deterioration [36].

Some studies reported dementia prevalence or incidence rather than specific test scores. Alaa A. showed increased dementia incidence over a 16-year period in diabetic PD patients [18], while M.I. Khalil identified T2DM as a significant risk factor for accelerated PDD onset [22]. Inflammatory pathways were also implicated; for instance, Saul Martínez-Horta et al. found elevated IL-2 and IL-6 levels in T2DM-PD patients with dementia, suggesting metabolic neuroinflammatory interactions [33].

Notably, a few studies found no significant association between metabolic status and cognition, such as E. Hogg et al., who reported no correlation between HOMA-IR and MoCA [23], and R. Wang et al., who observed no measurable cognitive changes despite IR [29]. These exceptions may reflect methodological differences, shorter follow-up durations, or population heterogeneity.

Overall, cognitive decline was more robustly and consistently observed in T2DM-PD than in IR-PD, with declines spanning global cognition, memory, and attention, with supporting evidence from neuroimaging, biomarker analyses, and longitudinal dementia incidence studies. While IR alone was less consistently detrimental, when present alongside PD, it often correlated with poorer outcomes in at least one cognitive domain. Detailed results are provided in Supplementary Tables S1 and S2 of the Supplementary Materials.

### 3.2. Study Quality and Potential Sources

**Figure 3:**
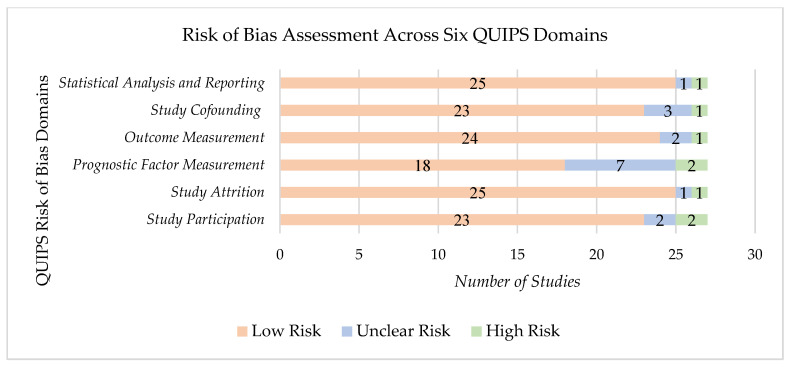
Graphs indicating risk of bias assessment across six QUIPS domains (27 studies).

The Quality in Prognostic Studies (QUIPS) tool was selected to assess the risk of bias in our studies, which were mostly observational studies, as it offers a six-domain-based evaluation tailored to examining biases in prognostic factor research; other tools were not chosen, as they were either too general or offered a focus on intervention-based exposures. QUIPS includes assessing confounders and outcome measurements, both essential for our review question [42]. A summary of the risk of bias assessment across all six QUIPS domains is shown in Figure 3.

Quality assessment revealed most studies had a low risk of bias due to well-defined selection criteria and consistent CD assessment methods. These studies adhered to standardized and validated tools for outcome measurement, which strengthened their reliability. However, two studies exhibited unclear risk of bias due to a lack of clarity regarding inclusion criteria [15,27]. Additionally, two studies had a high risk of bias because of small sample sizes [24,35].

Bohnen et al.’s study was identified as having a high risk of bias [24], particularly in the participation domain. The exclusive inclusion of Caucasian participants may have compromised the broader applicability of the findings, limiting the study’s external validity.

The “prognostic factor measurement” domain showed most instances of unclear risk of bias. A common issue across many studies was the lack of detailed laboratory data, particularly in relation to metabolic markers such as HbA1c or HOMA-IR. This caused difficulty in accurately assessing their role in the CD of PD patients, introducing some interpretation uncertainty.

### 3.3. Effect of Exposure on Outcome

#### 3.3.1. T2DM and IR

Study outcomes were assessed using HbA1c, HOMA-IR, and a range of cognitive tests. Among the 23 clinical studies, 15 reported that T2DM was a contributor to CD in patients with PD, while 4 studies specifically examined IR as a primary exposure (check Table 3 for more details). Elevated HbA1c was consistently associated with worse cognitive performance and a higher likelihood of PDD. For example, L. Yang et al. found that PD patients with T2DM were more likely to develop PDD than non-diabetic individuals [34]. MoCA scores were lower in patients with HbA1c ≥ 6.5% (24.41 ± 0.91 vs. 26.02 ± 2.21, *p* = 0.016), HbA1c ≥ 7% (24.96 ± 1.85 vs. 26.03 ± 2.21, *p* = 0.007), and HOMA-IR ≥ 3 (24.93 ± 1.58 vs. 25.94 ± 2.23, *p* = 0.049) compared with their respective controls. Elevated HbA1c ≥ 7% was also identified as an independent risk factor for cognitive impairment in PD, with logistic regression showing an odds ratio of 4.25 (95% CI: 1.59–11.34, *p* = 0.004) [27]. Other clinical studies similarly linked poorly controlled T2DM to accelerated cognitive deterioration, often mediated by vascular, inflammatory, and metabolic pathways. Most included a higher proportion of male participants, reflecting the known male predominance of PD and PDD. Ethnicity was predominantly Caucasian or Asian, limiting the generalizability of the findings.

Preclinical models reinforce these clinical observations. Zhang et al. showed that high-fat-diet (HFD) feeding for 22 weeks aggravated MPTP-induced dopaminergic loss by 38% compared with MPTP-only mice (*p* < 0.01) [38]. Hong et al. reported that T2DM (HFD + streptozotocin) increased HOMA-IR 2.1-fold and oxidative stress, leading to ~45% greater substantia nigra dopaminergic cell loss (*p* < 0.001) [39]. Morris et al. found that IR induced by HFD increased α-synuclein accumulation by 35% and reduced mitophagy [41], while Wang et al. observed that metabolic inflammation (HFD + low-dose streptozotocin) increased microglial activation 1.7-fold and exacerbated neurodegeneration by ~40% (*p* < 0.01) [40]. These findings highlight biological mechanisms, neuroinflammation, mitochondrial dysfunction, and α-synuclein aggregation through which T2DM and IR may worsen PD-related cognitive decline.

#### 3.3.2. Cognitive Decline (CD) and Parkinson’s Disease Dementia (PDD)

Among the 27 included studies, 9 focused primarily on CD without a formal diagnosis of dementia [16,19,24,28,30,31,34,36,41], while 8 specifically examined PDD [15,18,21,22,23,27,32,33]. The remaining studies evaluated mixed cognitive outcomes or reported domain-specific impairments such as executive function, attention, and memory.

Across the studies, cognitive impairment was consistently more pronounced in PD patients with T2DM or IR compared to those without these metabolic conditions. MoCA and MMSE scores were the most frequently used tools, reported in 17 of the 23 clinical studies (73.9%), showing lower average scores in PD patients with T2DM/IR. For example, Khalil et al. reported MoCA scores of 18.75 ± 4.4 in demented PD patients with T2DM versus 27 ± 1.85 in non-demented patients (*p* < 0.001) [22]. Similarly, Athauda et al. found significantly reduced MoCA scores (23.6 ± 0.3) in PD patients with T2DM compared to 25.0 ± 0.1 in non-diabetic PD patients (*p* < 0.001) [31].

Quantitative findings further support these patterns. Bohnen et al. observed a mean cognitive z-score of 0.98 ± 1.01 in PD patients with T2DM, indicating mild cognitive impairment, compared to −0.36 ± 0.91 in controls [24]. Qing Wang et al. reported that demented PD-T2DM patients had significantly higher LDL-C (*p* = 0.028), fibrinogen (*p* = 0.036), and hs-CRP (*p* = 0.017) levels, which are biomarkers frequently linked to IR and vascular inflammation [32].

Vascular, inflammatory, and metabolic dysfunction emerged as recurring contributors to cognitive deterioration. While most studies (*n* = 23) supported an association between T2DM/IR and worse cognitive outcomes, four studies reported no significant association, often due to small sample sizes, short follow-ups, or variability in diagnostic criteria.

A minority of studies (3/27) used neuroimaging to assess brain structural changes, with reduced gray matter volumes associated with poorer cognitive performance [27,34,40]. For example, Wang et al. reported mean MoCA scores of 9/30 in demented patients versus 22/30 in non-demented patients, with a diagnostic cutoff of ≤21 points [40].

Weighted averages across studies revealed MoCA and MMSE scores of approximately 23.8 and 27.4, respectively, in PD patients with T2DM/IR. Average gray matter volumes were around 519.34 mm^2^ in the subset of studies reporting volumetric data. These results indicate a robust but heterogeneous association between T2DM/IR and cognitive decline in PD, with the strongest effects observed in patients with higher metabolic burden and vascular risk profiles.

## 4. Discussion

### 4.1. Major Findings

In this review of 27 studies, 15 of the clinical studies reported that T2DM was associated with CD in PD, while 4 specifically examined IR as a primary exposure. The studies linked poor glycemic control (HbA1c ≥ 7%) and elevated HOMA-IR to lower MoCA/MMSE scores, greater prevalence of PDD, and accelerated cognitive deterioration. Neuroimaging data from three studies revealed reduced gray matter volume and increased white matter hyperintensity in PD patients with T2DM, correlating with poorer cognitive outcomes.

Vascular, inflammatory, and metabolic markers, including elevated LDL-C, fibrinogen, hs-CRP, and pro-inflammatory cytokines, were frequently reported alongside cognitive impairment, supporting their role as potential mediators.

Preclinical models provided mechanistic insights, showing that diet or chemically induced T2DM/IR exacerbated α-synuclein accumulation, mitochondrial dysfunction, neuroinflammation, and dopaminergic neuronal loss by 35–45% compared with controls.

Although most studies supported an association between T2DM/IR and worse cognitive outcomes, some reported no significant relationship, often due to small sample sizes, short follow-up periods, or variation in diagnostic criteria. Therefore, larger, multi-ethnic, longitudinal cohorts are needed to determine causality and inform targeted interventions.

### 4.2. Mechanisms and Pathophysiology Linking T2DM/IR to Cognitive Decline

#### 4.2.1. Insulin Resistance and Brain Function

Although not fully understood yet, several hypothesized mechanisms have been proposed to link cognitive decline to insulin resistance. Experimental studies have suggested that disruption in the insulin signaling pathway in the brain may contribute to neuroinflammatory cascades involving inflammation, oxidative stress, and increased a-synuclein deposition in the brain, all of which serve to accelerate neuronal death and are associated with poorer prognosis in PD [43,44,45].

**Figure 4:**
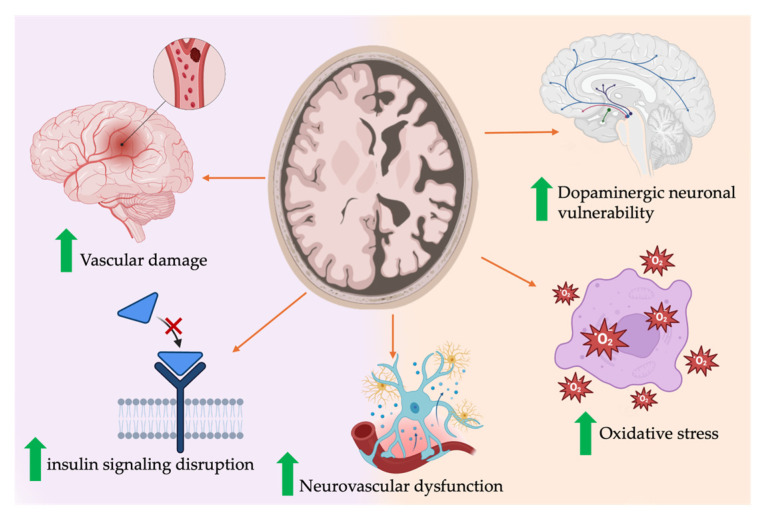
Mechanistic pathways linking T2DM/IR to cognitive decline in PD. Created with BioRender.com.

Furthermore, insulin resistance caused by glucose variability is thought to cause damage to cerebral small vessels, which could potentially hinder the clearance of pathogenic proteins in the brain, such as α-synuclein, resulting in aggregation with the development of cognitive decline, leading to dementia [39,46,47]. Notably, one study suggested that glucose variability, when compared to consistently hyperglycemic states, caused more damaging effects on inflammatory responses, endothelial dysfunction, and oxidative stress [32]. Insulin is also thought to have an important role in neurotransmission through its interaction with dopamine. Preclinical findings have proposed the possibility of insulin resistance exacerbating dopaminergic neuronal vulnerability and potentially accelerating CD in PD [48]. However, it is important to note that these proposed mechanisms remain largely theoretical and will require validation through further longitudinal and clinical studies. Figure 4 provides a visual summary of proposed mechanisms linking insulin resistance to cognitive decline in PD.

#### 4.2.2. T2DM

Many potential mechanisms linking type 2 diabetes mellitus to cognitive decline and dementia in PD have been proposed. T2DM has been suggested as a significant risk factor contributing to brain atrophy involving the cerebral cortex and limbic structures [49]. Furthermore, it is suggested that T2DM accelerates the aggregation of amyloid plaques and neurofibrillary tangles in the brain [50]. This provides a mechanistic link between hyperglycemia and the metabolic changes accompanied by T2DM and the exacerbation of neurodegenerative processes in PD. In addition, one study explored the potential of circulating neurotoxic 5-HT2A receptor agonist autoantibodies in PD patients with DM, with a link to cognitive dysfunction [15]. The presence of the 5-HT2A autoantibodies was suggested to accelerate neuronal loss, hypothetically through the activation of these receptors, causing the autoantibodies to induce neurite retraction. In addition, the study suggested that the presence of immunoglobulin (IgG) also contributes to the neurodegenerative process. Altered calcium homeostasis, such as alterations in the activity of Calpain I and expression of calcium-buffering proteins, may also play a role in the pathology [51]. Chronic inflammation is hypothesized to alter the expression of receptors, which may contribute to the mediation of vascular and neurotoxic effects.

#### 4.2.3. Vascular Contributions

Vascular inflammatory mediators are greatly expressed in PD patients with DM and dementia. Fibrinogen, an inflammatory mediator, is hypothesized to drive the process of neurodegeneration through activation of central nervous system (CNS) vascular inflammation, dysregulation of microcirculatory function, and disruption of the blood–brain barrier (BBB) [52] and neurovascular units (NVUs), hypothetically due to greater fragmentation of capillaries and chronic inflammatory damage in multiple brain regions, including the frontal cortex, hippocampus, and prefrontal lobe. This inflammation likely contributes to the acceleration of the cognitive decline in PD patients diagnosed with DM [53,54].

### 4.3. Limitations and Bias

Most of the included studies determined participants’ T2DM or IR status using objective biochemical measures such as HbA1c, the oral glucose tolerance test (OGTT), fasting blood/plasma glucose (FBG/FPG), or fasting insulin (FINS), which minimizes misclassification bias. However, a notable limitation was that 4 of the 27 included studies relied on self-reported diagnosis [17,24,30,31]. Self-reporting is prone to underestimation in undiagnosed individuals; global estimates suggest that up to 38–46% of T2DM cases remain undiagnosed, with underreporting among those already diagnosed [55]. These issues are further amplified by recall inaccuracies, especially in older adults or those with mild cognitive impairment, and can lead to misclassification into control groups. This not only reduces the size of exposed groups but also likely biases effect estimates toward the null.

**Figure 5:**
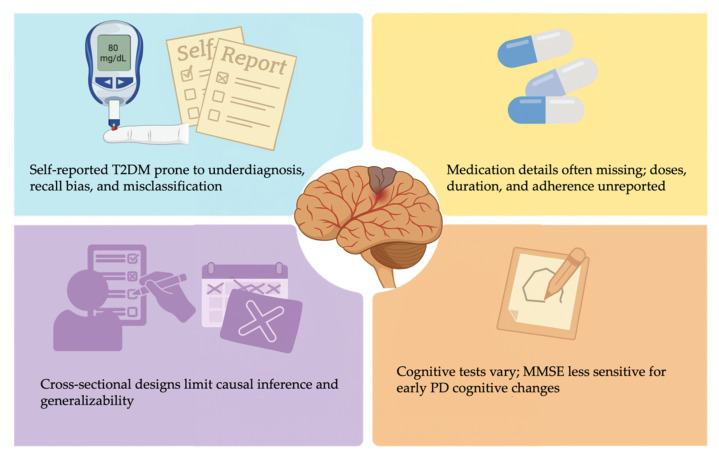
Key limitations in included studies on T2DM/IR and cognitive decline in PD.

Another important limitation is the lack of systematic reporting on anti-diabetic and anti-Parkinsonian medications. Only 5 of the 27 studies [20,24,30,31,35] reported any details on medication use, and even these lacked dosing, duration, or adherence data. Pharmacological interventions can directly influence both PD progression and cognitive outcomes, thus confounding the observed association between T2DM/IR and cognitive decline. Figure 5 provides a visual summary of the key methodological limitations.

Metformin has demonstrated dose-dependent neuroprotection in preclinical PD models via AMPK activation, reduced α-synuclein phosphorylation, and improved mitochondrial function [56,57,58]. While some clinical studies associate metformin with slower PD progression, others report no effect, potentially due to dose or duration differences [56]. Insulin therapy, by improving metabolic control, has also been linked to slower PD progression and cognitive decline [59]. By contrast, sulfonylureas show mixed evidence, with some in vivo neuroprotective effects but inconsistent clinical results, with suggestions of possible PD acceleration [56,60]. GLP-1 receptor agonists, which may inhibit α-synuclein aggregation, have shown early cognitive benefits in Phase II trials, though they are not yet approved for clinical use [61,62,63]. Therefore, the absence of these medication details in most studies prevents adjustment for these potentially confounding effects.

Study design also imposes limitations. In total, 17 of the 27 studies were cross-sectional (Table 3), which limits causal inference and the ability to establish temporal relationships between T2DM/IR and cognitive decline or PDD. Ethnic representation was skewed toward Caucasian and Asian populations, restricting applicability to other ethnic groups.

Cognitive assessment heterogeneity is another major limitation. While 20 studies used the MoCA and/or the MMSE, others used different tools such as the Brief Cognitive Battery (BBRC-Edu) [35]. The MMSE, although widely used, lacks sensitivity for detecting mild cognitive impairment (MCI) and executive dysfunction, both common in PD, and is strongly influenced by age and educational attainment, which can cause misclassification, especially in elderly T2DM cohorts [64]. MoCA is generally more sensitive than MMSE for detecting early cognitive changes in PD, particularly in executive and visuospatial domains [65,66]. However, MoCA performance varies with education level, language, and cutoff thresholds [67], complicating cross-study comparability.

These measurement heterogeneity differences in domain coverage, sensitivity, and diagnostic thresholds introduce risk of misclassification and reduce comparability across studies. Moreover, some studies defined cognitive outcomes as “presence of cognitive decline,” while others used “onset of dementia,” creating further difficulty in comparing exposure and control groups directly.

### 4.4. Comparison with the Literature and Clinical Implications

Our findings align with prior observational and mechanistic studies linking T2DM, IR, and CD in PD [24,31,34]. While earlier research has reported associations between metabolic dysfunction and deficits in memory and executive function, no systematic review has synthesized both clinical and preclinical evidence on this topic. By integrating results from 27 studies, our review provides an overview of the consistency and potential biological mechanisms underlying this association.

Clinically, these results show that early detection and management of T2DM/IR in PD patients could help prevent or delay CD and the onset of PDD. Metabolic assessments such as HbA1c and HOMA-IR may offer valuable tools for risk stratification in PD. For instance, Uyar et al. found that elevated HbA1c in PD patients was associated with increased neuroaxonal damage markers like neurofilament light chain and lower MoCA scores, independent of age, vascular risk factors, and BMI [68].

Although current evidence does not confirm that metabolic monitoring or targeted treatment modifies the trajectory of CD or delays PDD onset, these approaches merit further investigation. Future research should clarify the predictive value of metabolic measures and evaluate their integration into PD management strategies.

### 4.5. Conclusions

Our review concludes a possible association between T2DM and/or IR and CD in patients with PD. Most included studies highlight the critical role of T2DM and IR in cognitive impairment and PDD. However, our findings underscore the necessity for larger sample sizes and controlling for clinical variables, such as HbA1c levels and pharmacological interventions, which could provide deeper insights into the relationship between metabolic dysfunction and CD. To further substantiate these associations, longitudinal studies with systematic follow-ups are essential to clarify the causal relationship and underlying mechanisms. There is a clear need to explore additional biomarkers that could predict cognitive outcomes in PD patients with T2DM/IR. These biomarkers could serve as early indicators for CD, improving the management of PD patients with metabolic disturbances.

## Figures and Tables

**Table 1:** Inclusion and exclusion criteria.

PECO Component	Inclusion Criteria	Exclusion Criteria
Population (P)	1—Adults (>18 years) diagnosed with PD, with or without a documented diagnosis of T2DM or IR. 2—Studies assessing cognitive outcomes specifically in PD patients with known T2DM or IR.	Studies on populations without a PD diagnosis or focusing on other neurodegenerative disorders.
Exposure (E)	1—Studies examining the impact of DM and IR on cognitive function in PD patients. 2—Observational studies that specifically measure IR (using, e.g., HOMA-IR, HbA1c) and non-human populations.	1—Studies not specifically analyzing T2DM or IR in the context of PD. 2—Research focused exclusively on other metabolic disorders without any connection to T2DM or IR.
Comparison (C)	1—Comparisons between PD patients with T2DM or IR versus those without these metabolic conditions. 2—Comparative studies examining cognitive outcomes between PD populations stratified by T2DM status, IR levels, or glycemic control.	1—Studies lacking a comparison group related to metabolic conditions or without cognitive assessment data.
Outcome (O)	1—Cognitive outcomes measured by validated cognitive scales (e.g., MMSE, MOCA) in PD patients. 2—Diagnosis or evidence of dementia, or CD specifically attributed to PD.	1—Studies that do not measure cognitive outcomes. 2—Outcomes unrelated to CD or dementia.
Study Characteristics	1—Primary articles. 2—Case–control studies. 3—Original works written exclusively in English. 4—Studies published in the last 20 years (>2005). 5—Preclinical/clinical studies.	1—Editorials. 2—Letters. 3—Expert opinion.

PD: Parkinson’s disease. T2DM: type 2 diabetes mellitus. IR: insulin resistance. HOMA-IR: Homeostatic Model Assessment for Insulin Resistance. HbA1c: hemoglobin A1c. MMSE: Mini-Mental State Examination. MoCA: Montreal Cognitive Assessment.

**Table 2:** Study range and characteristics.

Characteristics	Details
Study Designs	Cross-sectional (*n* = 15) Retrospective (*n* = 6) Prospective (*n* = 2) Preclinical (*n* = 4)
Sample Size	Majority: 73–1930 participants Smaller clinical studies (2): 23–36 participants Larger preclinical studies (2): 147,096–544,162 participants
Age Range	Mean age: 59–79 years Most studies focused on older adults (60–75 years)
Gender Distribution	Across all studies, a larger proportion of participants were males
Ethnicity	Several studies had primarily Asian populations (Chinese, Japanese, Korean, Bangladeshi, etc.)

**Table 3:** Summary of clinical and preclinical studies evaluating the association between insulin resistance/type 2 diabetes mellitus and cognitive decline or dementia in Parkinson’s disease.

Clinical Studies					
Author, Year, Country	Population	Exposure	Measurement Tool	Main Cognitive Outcome	Key Findings
Alaa A., 2020, (UK) [18]	- Total N = 544,162 - Age: 73.1 - Male 41.9%, Female 58.1%	T2DM	N/A	139/544,162 PDD	Increase in the prevalence and incidence of dementia in diabetic individuals from 2000 to 2016.
J. Park, 2023 [19]	- Total N = 262 - Age: 71.1 - 57% male, 43% Female	T2DM and Prediabetes	Diabetes (HbA1c ≥ 6.5); prediabetes (HbA1c 5.7–6.4%)	CD	Prediabetes and diabetes were associated with worse MoCA scores in PD patients (*p* = 0.002).
Miyake Y., 2010, Japan [20]	- N = PD 249, non-PD 368 - Age: 69.1 ± 8.4 - Gender: N/A	T2DM	Structured questionnaire	These vascular risk factors had a significantly lower risk of developing PD	The study found inverse associations between vascular/metabolic diseases and PD.
Seong-Beom Koh, 2024, South Korea [21]	- Total N = 9264 - Age: 71.3 ± 8.45 - Gender: N/A	T2DM	HBA1c (%)	1757/9264 PDD	Compared to consistent hyperglycemia, glucose variability is noted to have more deleterious effects on inflammation.
M. Ibrahim Khalil, 2021, Bangladesh [22]	- Total N = 131 - Age: 73.32 ± 8.86 - Gender: N/A	T2DM	FBG ≥ 7.0 mmol/L and/or 2 h post	17/29 PDD	Diabetes was identified as a significant risk factor for the exacerbation of cognitive decline.
E. Hogg, 2018, USA [23]	- Total N = 160 - Age: 67.7 ± 10.5 - Males: 109, Females: 51	IR	HOMA-IR	90/160 PDD	No significant correlation was found between HOMA-IR and MoCA, MDS-UPDRS.
N. Bohnen, 2014, USA [24]	- Total N = 148 - Age: 67.3 ± 6.1 - 109 Males, 39 Females	T2DM	Self-reported	CD	The study observed that PD patients with DM showed the greatest impairments in attentional function.
Bosco D., 2012, Italy [25]	- Total N = 110 - Age: 65 ± 6.2 - 72 Males, 38 Females	IR	2-h OGTT, HOMA-index, MMSE, UPDRS, MADRS	IR is strongly associated with dementia in PD	IR correlated with lower MMSE scores.
I. Markaki, 2021, Sweden [26]	- Total N = 244 - Age: 64 - 64% Male, 36% Female	T2DM	HBA1c (%)	N/A	Cognitive decline was observed with imbalances in HbA1c levels, but associations were not statistically significant.
M. Ong, 2017, Singapore [27]	- Total N = 77 - Age: 64.4 ± 7.62 - 76.9% Males, 23.1% Females	T2DM	HBA1c or FBG ≥ 7	12/77 PDD	The study provides evidence that PD patients with DM exhibit lower gray matter volume
M. Giuntini, 2014 [28]	- Total N = 100 - Age: 64.46 ± 6.72 - 56% Male, 44% Female	T2DM	American Diabetes Association criteria for DM	CD	The presence of DM in PD has a negative impact on the progression of PD.
R. Wang, 2024, China [29]	- Total N = 73 - Age: 61.68 ± 7.45 - 61.3% Female, 38.7% Male	IR	HOMA-IR: Median: 2.68 (1.56, 3.41)	N/A	The study found no significant change in cognitive function.
Jolie D. Barter, 2023 [30]	- Total N = 424 - Age: 69.94 ± 7.6 - 83.3% Male, 16.7% Female	T2DM	Self-report + fasting glucose	CD	Significant interaction effects (*p* < 0.01) for Stroop Interference and logical memory recall.
N. Palacios, 2011, USA [17]	- Total N = 147,096 - Age = 63.6, - Gender: N/A	T2DM	Self-reported	N/A	Contrasts with some prior studies reporting increased risk.
Dr. Dilan Athauda, 2022, UK [31]	- Total N = 1930 - Age: 71.1 (0.7) - 72.5% Male, 27.5% Female	T2DM	Self-reported	CD	After controlling for confounders, findings indicated that PD patients with T2DM had more severe symptoms.
Qing Wang, 2020, China [32]	- Total N = 928 - Age: 79.0 - Gender: N/A	T2DM	HBA1c (%)	31/215 PDD	The study identified that lower levels of LDL-C and higher levels of fibrinogen were associated with more severe CD in PD patients with T2DM.
Saul Martínez-Horta, 2021, Spain [33]	- Total N = 533 - Age: 67.1 ± 6.4 - Gender: Male % higher	T2DM	N/A	PDD	The study found that IL-2 and IL-6 levels were higher in patients with PDD and DM.
L. Yang, 2017, China [34]	- Total N = 282 - Age: 70.79 ± 7.63 - 51.8% males, 48.2% Females	T2DM	HBA1c (%)	CD	Regression analysis showed a significant negative correlation between MoCA scores and both HbA1c and IR.
Arthur Oscar Schelp, 2017, Brazil [35]	- Total N = 142 - Age: 73.85 ± 6.62 - 64.4% Male, 35.6% Female	IR	HOMA-IR	CD + PDD	IR, older age, and lower education levels correlated with poorer memory performance.
Brit Mollenhaur, 2019, Germany [36]	- Total N = 135 - Age: 64.55 ± 9.84 - Gender: N/A	T2DM	HBA1c (%)	CD	Specific biomarkers, such as elevated periodic limb movement index during sleep, led to cognitive decline.
Eduardo de Pablo-Fernández, 2021, UK [37]	- Total N = 132 - Age: 70.4 ± 8.1 - 45% Male, 55% Female	T2DM	FPG ≥ 126 mg/dL	N/A	The study provides evidence that PD with T2DM shows faster disease progression.
M. Petrou, 2016, USA [16]	- Total N = 36 - Age: 66.0 ± 5.2 - 83.3% Male, 16.7% Female	T2DM	N/A	CD	The study provides evidence that diabetes is associated with greater gray matter loss in PD patients.
M. Zimering, 2018, USA [15]	- Total N = 23 - Age: 70.8 ± 5.6 - Gender: older adult males	T2DM	HBA1c (%)	1/10 PDD	The study showed that mean accelerated neuroblastoma cell loss was induced by diabetic Parkinson’s disease.
Preclinical Studies					
Zhang et al., 2024, China [38]	- Total N = N/A - Age: 4-week-old mice - Gender: 100% Male	T2DM	Blood glucose levels	T2DM exacerbated the motor and cognitive symptoms in PD mice	T2DM microenvironment significantly exacerbates PD pathology, primarily through mitochondrial dysfunction.
Hong et al., 2020, China [39]	- Total N = 12 - Age: N/A - Gender: Male rats	IR + MPTP	HOMA-IR	Dopaminergic neuron loss and oxidative stress (exacerbated)	IR promotes PD pathology by disrupting PLK2 signaling and mitochondrial function.
Wang et al., 2014, China [40]	- Total N = 40 mice - Age: 10–12 weeks - Gender: Male	T2DM (HFD + low-dose STZ)	Fasting glucose, insulin levels, inflammatory markers	Dopaminergic neuronal degeneration (exacerbated)	T2DM-induced metabolic inflammation amplifies susceptibility to dopaminergic neuron loss.
Morris et al., 2010, USA [41]	- N = 4 - Age: N/A - Gender: Male mice	HFD (IR model)	Dopaminergic neuron count (SNpc), tyrosine hydroxylase staining	CD	HFD-induced IR worsens dopaminergic neuron vulnerability in PD models.

Abbreviations: Total N: total number, HbA1c: glycated hemoglobin, HOMA-IR: Homeostatic Model Assessment of Insulin Resistance, MDS-UPDRS: Movement Disorder Society—Unified Parkinson’s Disease Rating Scale, OGTT: oral glucose tolerance test, UPDRS: Unified Parkinson’s Disease Rating Scale, MADRS: Montgomery–Åsberg Depression Rating Scale, FBG: fasting blood glucose, 5-HT2A: 5-Hydroxytryptamine 2 LDL-C: low-density lipoprotein cholesterol, IL: interleukin; FPG: fasting plasma glucose, HFD: high-fat diet, STZ: streptozotocin, SNpc: substantia nigra pars compacta, PLK2: Polo-Like Kinase 2.

## Data Availability

All data are contained within the manuscript.

## Supplementary Materials

The following supporting information can be downloaded at: https://www.mdpi.com/article/10.3390/ijms26168078/s1.
