# Supplementary material for: The Influence of Insulin Resistance and Type 2 Diabetes on Cognitive Decline and Dementia in Parkinson’s Disease: A Systematic Review
Source: Int J Mol Sci. 2025 Aug 21;26(16):8078. doi: 10.3390/ijms26168078 (PMC12386517; doi:10.3390/ijms26168078)
Supplement: Supplementary file 1 [file ijms-26-08078-s001.zip › ijms-3778795-supplementary.pdf]

Supplementary Table S1: Summary of Included Studies Examining the Impact of Type 2 Diabetes Mellitus (T2DM) and Insulin Resistance (IR) on Cognitive Decline (CD) and Parkinson's Disease Dementia (PDD) and Reported Pathophysiological Associations

A comprehensive summary of the 17 studies included in this systematic review, detailing their study design, objectives, sample characteristics, exposure definitions, measurement tools, outcomes, and proposed mechanisms linking T2DM or IR with cognitive impairment in Parkinson's disease. Studies are arranged chronologically and geographically to illustrate global research efforts across diverse populations.

	Study Characteristics			Population					Findings	
#	Author, Year, Country	Study Design	Study Aim	Age (years)	Gender	Sample Size	Exposure	Measurement Tool	Results	Outcomes
1	M.Ibrahim Khalil,2021, Bangladesh	Cross-sectional	To assess the association between type 2 diabetes mellitus (T2DM) and the development of dementia in Parkinson's disease patients, using longitudinal national health data to examine the effects	Demented PD cases: 73.32 ±8.86 non-demented cases: 63.98 ±6.19 (for total sample size not only PD-DM patients.)	out of 131 total sample size Demented: 26 Male, 24 Female<br><br>Non-demented: 55 Male, 26 Female	131	T2DM	FBG ≥7.0 mmol/L and/or 2-h post-load glucose concentration of ≥11.1 mmol/Lor currently receiving T2DM medication	Not mentioned	17/29 PDD

			of glucose variability on dementia risk.							
2	M. Ong, 2017, Singapore	Prospective cohort	To evaluate the influence of diabetes mellitus on longitudinal brain atrophy and cognitive decline in Parkinson's disease patients.	64.4 ± 7.62	M 50 (76.9%)	77	T2DM	HBA1c or FBG ≥7	Not mentioned	12/77 PDD
3	E. Hogg, 2018, USA	Cross-sectional	To determine the prevalence of insulin resistance in non-diabetic Parkinson's disease patients and investigate its association with metabolic indicators, motor/non-motor symptoms, and quality of life.	67.7 ± 10.5 years	Males: 109 Mean ± STD: 2.4 ± 1.9. Females: 45 Mean ± STD: 2.0 ± 1.2	160	IR	HOMA-IR	HOMA-IR= 2.3 ± 1.8	90/160 PDD
4			To investigate the association between glycated hemoglobin	64 (12)	36% Female	244	T2DM	HBA1c (%)	244 PD patients 184 (75.4%) were euglycemic, 17 (7%) had low	N/A

	I.Markaki, 2021, Sweden	Longitudinal Observational	(HbA1c) levels and motor and cognitive symptom progression in Parkinson's disease, evaluating how glycemic control (both high and low HbA1c) influences outcomes over time						and 18 (7.4%) had high HbA1c, and 25 (10.2%) had DM.	
5	Seong-Beom Koh,2024, South Korea	Retrospective cohort	To investigate whether visit-to-visit fasting glucose variability is associated with the development of Parkinson's disease dementia over a 9-year follow-up period using nationwide cohort data .	Demented PD cases: 73.32(±8.86) non-demented cases: 63.98(±6.19) (for total sample size not only PD-DM patients.)	out of 131 total sample size Demented: 26 Male 24 Female Non-demented: 55 Male 26 Female	9,264	T2DM	HBA1c (%)	HbA1c: 6.8 ± 1.2%	1,757/9,264 (19%) PDD
6	N.Bohnen, 2014, USA	Cross-sectional	To investigate whether diabetes mellitus is independently associated with more severe cognitive impairment in Parkinson's disease, after controlling for cholinergic and dopaminergic denervation using PET imaging.	67.3±6.1	86.7% Male, 109 M 39 F	148	T2DM	(self-report)	N/A	CD

7	Dr.Dilan Athauda, 2022, United Kingdom	Cross-sectional	To examine the impact of type 2 diabetes mellitus on Parkinson's disease progression, including both motor and non-motor symptoms, using data from a large cohort (Tracking Parkinson's study) .	71.1 (0.7) (total sample)	72.5% Male	1,930	T2DM	(self-report)	N/A	CD
8	L.Yang, 2017, China	Cross-sectional	To explore the association between cognitive impairment and dysglycemia (including diabetes and insulin resistance) in patients with Parkinson's disease, and to identify relevant biomarkers.	70.79 ± 7.63 (total)	51.8% males	282	T2DM	HBA1c (%)	HbA1c ≥ 6.5% for 29 patients, HbA1c < 6.5% for 90 patients	CD
9	Arthur Oscar Schelp, 2017, Brazil	Cross-sectional	To assess the relationship between cognitive impairment in Parkinson's disease and factors like body composition, ageing, and insulin resistance .	73.85±6.62	M and F (Overall: Male: 64.4%, Female: 35.6%)	142	IR	HOMA-IR	HOMA-IR: 19.57 ± 7.21	CD + PDD
10	Alaa A., 2020, (UK)	Retrospective cohort	To estimate the prevalence and incidence of dementia among people with diabetes	DM-D (of total sample size 28,772) =	Male= 12,064 (41.9)	544,162	T2DM	N/A	N/A	139/544,162 PDD

			in the UK, using data from The Health Improvement Network (THIN) primary care database .	1. mean age at first DM diagnosis: 73.1 years (10.0) 2. mean age at first dementia diagnosis: 80.4 years (7.9)	Female= 16,708(58.1)					
1 1	M. Zimering, 2018, USA	Retrospectiv e cohort	To test for the presence of circulating neurotoxic 5-HT2A receptor agonist autoantibodies in adults with type 2 diabetes and Parkinson's disease, and to investigate their potential role in the pathogenesis of neurodegeneration.	70.8 ± 5.6	older adult males	23	T2DM	HBA1c (%)	7.8 ± 1.4% "	1/10 PDD
			To investigate how hyperglycemia affects motor symptoms, particularly axial	61.68 ± 7.45	Female 19 (61.3%) and Male 12 (38.7%)	73	IR	HOMA-IR: Median: 2.68 (1.56, 3.41)	CD + PDD	Circulating Neurotoxic 5-HT2A Receptor Agonist Autoantibodies in Adult Type 2 Diabetes with Parkinson's

12	R. Wang, 2023, China	Cross-sectional	signs, in Parkinson's disease, and to explore insulin resistance-dependent and independent mechanisms							Disease Veterans Affairs New Jersey Healthcare System (VANJHCS), East Orange, NJ
13	Eduardo de Pablo-Fernández, 2021, United Kingdom	Retrospectiv e cohort	To examine whether faster disease progression in Parkinson's disease patients with type 2 diabetes is related to increased neuropathology, including α-synuclein, tau, amyloid-β, or vascular pathology	70.4 ± 8.1	45% Male	132	T2DM	FPG ≥126 mg/dL or diagnosis of T2DM by treating primary care physician or regular treatment with hypoglycemic medication.	Not mentioned	N/A
14	Brit Mollenhaur , 2018, Germany	Prospective cohort	To identify baseline predictors associated with disease progression, including cognitive decline, over a 4-year period in early Parkinson's disease patients using data from the DeNoPa cohort.	64.55 ± 9.84	Female mean = 0.348 (0.478)	135	T2DM	HBA1c (%)	HbA1c, %= 5.326 (0.522)	CD
			To investigate the prevalence and	Demented: 79.0(72.0,84.0) non-demented:	Not provided	928	T2DM	HBA1c (%)	D+: 6.4(6.0,7.4) D-: 8.0(7.1,9.0)	31/215 PDD

15	Qing Wang, 2020, China	Retrospective cohort	contribution of vascular, inflammatory, and metabolic risk factors to the development of dementia in Parkinson's disease patients with type 2 diabetes mellitus, using a case–control design to explore modifiable contributors to cognitive decline.	73.0(66.0,80.0)						
16	Saul Martínez-Horta,2021, Spain	Cross-sectional	To identify comorbidities and lifestyle factors that contribute to cognitive profiles in early Parkinson's disease patients, aiming to understand the heterogeneity in cognitive outcomes	PD-MCI: 67.1 ± 6.4; PDD: 68.1 ± 8.6 (but not stratified by DM)	Proportion not specified. Male % higher.	533	T2DM	Not reported	N/A	PDD
17	M.Petrou, 2016, USA	Cross-sectional	To investigate effects of comorbid diabetes mellitus on cognition in Parkinson's disease, independently of disease-specific neurodegenerative changes.	66.0 ± 5.2	83.3% male	36	T2DM	Not reported	N/A	CD
18	J. Park, 2023	Cross-sectional	To assess the association between prediabetes/diabetes and cognitive function in PD patients	Diabetes: 71.1 (8.0) Prediabetes: 69.2 (8.3) Non-diabetic: 69.8 (10.3)	Diabetes: 57% male, Prediabetes: 44% male, Non-diabetic: 51% male	Total: 262 Diabetes: 76 Prediabetes: 90 Non-diabetic: 96	T2DM and Prediabetes	Diabetes (HbA1c ≥ 6.5); prediabetes	Diabetes: 17.0 ± 6.6. Prediabetes: 18.0 ± 6.1. Non-diabetic: 20.0 ± 5.7	CD

								(HbA1c 5.7-6.4%)		
19	M. Giuntini, 2014	Cohort Study	investigate potential differences in clinical presentation and progression between PD-DM patients with respect to PD-no DM patients.	PD-DM: 64.46 ± 6.72 PD – no DM: 64.96 ± 6.33	PD-DM: 56% male PD-no DM: N/A	PD-DM: 50 PD-no DM: 50	T2DM	Self-report + fasting glucose	PD+DM: 24 ± 4.7	CD
20	Wang et al., 2014, China	Preclinical (animal model)	To investigate how metabolic inflammation in type 2 diabetes mellitus (T2DM) mice exacerbates dopaminergic neurodegeneration following MPTP-induced Parkinsonism	10–12 weeks	Male	40 mice (10 per group: Control, T2DM, MPTP, T2DM+MPTP)	T2DM (HFD + low-dose STZ)	Fasting glucose, insulin levels, inflammatory markers	T2DM+MPTP mice showed significantly greater dopaminergic neuron loss in the substantia nigra, elevated microglial activation, increased IL-6, TNF-α, and oxidative stress markers compared to MPTP alone	Dopaminergic neuronal degeneration (exacerbated)
21	Jolie D. Barter, 2023	Cross-sectional	if individuals with comorbid PD and DM experienced poorer functional ability compared to individuals with only PD or DM	69.94 ± 7.6	men (83.3), women 6 (16.7)	424 individuals: healthy older adults (HOA), n = 170; PD without DM (PD-only), n = 162; DM without PD (DM-only), n = 56; and comorbid PD and DM (PD+DM), n = 36.	T2DM	Self-report + fasting glucose	PD+DM: 24 ± 4.7	CD

22	Hong et al., 2020, China	Preclinical (in vivo + in vitro)	To investigate how insulin resistance contributes to Parkinson's disease pathogenesis through α-synuclein expression, mitochondrial dysfunction, and PLK2 pathway deregulation	N/A	Male rats	12	IR + MPTP	HOMA-IR, TH immunostaining, ROS assays, Western blotting for PLK2 and α-synuclein	IR increased vulnerability to MPTP-induced damage, elevated α-synuclein aggregation, decreased mitochondrial membrane potential, increased ROS, and altered PLK2 signaling	Dopaminergic neuron loss, oxidative stress, protein aggregation
23	Morris et al., 2010, USA	Preclinical (mouse)	To determine whether a high-fat diet (HFD), modeling T2DM, worsens dopaminergic degeneration in MPTP-induced PD	N/A	Male rats	4 groups: Control diet + saline, HFD + saline, Control + MPTP, HFD + MPTP	HFD (IR model)	Dopaminergic neuron count (SNpc), tyrosine hydroxylase staining	HFD + MPTP group showed significantly greater dopaminergic neuronal loss vs MPTP alone	CD
24	Bosco D., 2012, Italy	Case-control study	To investigate glucose metabolism abnormalities and insulin resistance in PD patients with and without dementia.	PD-D: 65 ± 6.2 PD: 64.2 ± 6.3	PD-D: 36M / 17F PD: 36M / 21F	110 (53 PD-D, 57 PD)	IR	2-h OGTT, HOMA-index, MMSE, UPDRS, MADRS	PD-D had significantly higher insulin resistance (62%) than PD (35%). HOMA-index and 2h OGTT were significantly higher in PD-D.	Insulin resistance is strongly associated with dementia in PD even after adjusting confounders.
25	Zhang et al., 2024, China	In vivo mouse models and in vitro cell modes	To confirm the specific association between T2DM and PD through a co-morbid mouse model and cell model.	4-week-old mice.	100% Male	N/A	T2DM	Blood glucose levels.	> 13.9 mmol/L	T2DM exacerbated the motor and cognitive symptoms in PD mice.

26	Miyake Y., 2010, Japan	Case-control	To assess whether history of hypertension, hypercholesterolemia, and diabetes is associated with the risk of developing PD in a Japanese population.	PD 69.1 ± 8.4 non-PD 69.0 ± 8.5	N/A	PD 249 non-PD 368	Self-reported history of hypertension, hypercholesterolemia, or diabetes.	Structured questionnaire including medical history, medication use, and lifestyle factors.	Hypertension (OR: 0.43), hypercholesterolemia (OR: 0.58), and diabetes (Or: 0.38) were all inversely associated with PD risk.	Findings suggest that individuals in the Japanese population with these vascular risk factors had significantly lower risk of developing PD.
27	Natalia Palacios et al., 2011, USA	Prospective cohort study	To investigate whether obesity and diabetes are related to risk of Parkinson's disease.	Baseline: Men 63.6, Women 62.0; Mean age at PD onset: Men 71.9 (56.9–93.9), Women 71.2 (55.0–88.0)	63,303 men, 79,949 women	147,096	BMI, waist circumference, weight change, central vs. peripheral weight gain, baseline diabetes	Self-reported weight, height, waist circumference, diabetes diagnosis; BMI calculated	No significant association between baseline BMI, BMI at age 18, waist circumference, weight change, central weight gain, or baseline diabetes and PD risk. RR for diabetes at baseline: 0.88 (95% CI: 0.62–1.25, P=0.40).	PD diagnosis confirmed via treating neurologist/medical records; 656 incident PD cases confirmed during follow-up.

Abbreviations: PD – Parkinson's disease; T2DM – Type 2 diabetes mellitus; IR – Insulin resistance; CD – Cognitive decline; PDD – Parkinson's disease dementia; MoCA – Montreal Cognitive Assessment; MMSE – Mini-Mental State Examination; HbA1c – Glycated hemoglobin; HOMA-IR – Homeostatic Model Assessment for Insulin Resistance; FBG – Fasting blood glucose; N/A – Not available; MCI – Mild cognitive impairment.

Supplementary Table S2. Cognitive Outcomes in Parkinson's Disease Patients with Type 2 Diabetes Mellitus (T2DM) or Insulin Resistance

(IR) Linking T2DM and IR to Cognitive Outcomes in Parkinson's Disease

A summary of the the results from studies evaluating cognitive performance in PD patients with and without T2DM or IR. It includes cognitive assessments using the Montreal Cognitive Assessment (MoCA), Mini-Mental State Examination (MMSE), and brain gray matter volume

measurements, along with reported cutoffs, means, standard deviations, and p-values where available. The number of patients diagnosed with cognitive decline (CD) or Parkinson's disease dementia (PDD) is also shown for the intervention group.

					Results for Cognitive Decline Measurement									
		Total N of participants in intervention group (presence of DM/IR with PD)	Total N of participants in Control group (PD without DM)	Presence of cognitive decline Or Dementia (Y/N)	MoCA			MMSE			Gray Matter Volume			Number of patients with PDD or CD (Intervention Group)
#	Author, Year, Country				Result + cutoff	Mean ± SD	P-value	Result + cutoff	Mean ± SD	P-value	Result + cutoff	Mean ± SD	P-value	
1	M.Ibrahim Khalil,2021, Bangladesh	50	81	Y (CD)	N/A	N/A	N/A	N/A	Mean (SD): 18.75 ± 4.4 (Dementia) vs 27 ± 1.85 (non-dementia)	N/A	N/A	N/A	<0.001	17 (PDD)
2	M. Ong, 2017, Singapore	12	65	Y (CD)	N/A	Baseline: 26.75 ± 1.66. Change: −3.29 ± 3.68	0.016	N/A	Baseline: 28.75 ± 1.14. End: 26.46 ± 1.76	0.024	Lower volume – greater CI	Baseline: 480.26 ± 28.55. Change: −3.95 ± 9.94	0.665	11 (CD)
3	E. Hogg, 2018, USA	10	8	Y (Dementia)	N/A	N/A	N/A	N/A	N/A	N/A	N/A	N/A	N/A	2 (PDD)

4	I.Markaki, 2021, Sweden	25	219	Y (Dementia)	N/A	N/A	N/A	N/A	N/A	N/A	N/A	N/A	N/A	N/A
5	Seong-Beom Koh,2024, South Korea	9000	N/A	Y (Dementia)	N/A	N/A	N/A	N/A	N/A	N/A	N/A	N/A	N/A	1,757 (PDD)
6	N.Bohnen, 2014, USA	15	133	Y (CD)	Global Cognitive Z-Score. Not MOCA	0.98±1.01	0.006	N/A	N/A	N/A	N/A	N/A	N/A	15 (CD)
7	Dr.Dilan Athauda, 2022, United Kingdom	167	1763	Y (CD)	CI <26	23.6±0.3	<0.001	N/A	N/A	N/A	N/A	N/A	N/A	40 (PDD), 55 (CD)
8	L. Yang, 2017, China	85	197	Y (CD)	<26	23.35 ± 3.41	<0.001	N/A	27.32 ± 2.28	0.003	N/A	N/A	N/A	63 (CD)
9	Arthur Oscar Schelp, 2017, Brazil	47	75	Y (CD)	N/A	BBRC 75.1 +/- 4	N/A	N/A	N/A	N/A	N/A	N/A	N/A	47 (CD)
10	Alaa A., 2020, United Kingdom (UK)	350314	N/A	Y (CD)	N/A	N/A	N/A	N/A	N/A	N/A	N/A	N/A	N/A	139 (PDD)

11	M. Zimering, 2018, USA	71	64	Y (CD)	<22 consistent with dementia	26.4 ± 3.0	N/A, but the authors mention that relation is not statistically significant	N/A	N/A	N/A	N/A	N/A	N/A	5 (PDD), 23 (CD)
12	R. Wang, 2023, China	31	42	Y (CD)	N/A	N/A	N/A	Patients with illiteracy, primary education, or more than a junior education were identified as having CI when MMSE was below 17, 20, or 24 points, respectively	Median 28.00 (26.00, 29.00)	0.382	N/A	N/A	N/A	N/A
13	Eduardo de Pablo-Fernández, 2021, United Kingdom	25	107	Y (Dementia)	N/A	N/A	N/A	N/A	N/A	N/A	N/A	N/A	N/A	16 (PDD)
14	Brit Mollenhaur, 2018, Germany	135	109	Y (CD)	N/A	N/A	N/A	N/A	28.440±1.401	0.009	N/A	N/A	N/A	15 (CD)

15	Qing Wang, 2020, China	215	341	Y (CD)	N/A	D+: 9.0(8.0,13.0) D-: 22(20,24)	<0.001	N/A	D+: 13.0(11.0,17.0) D-:26.0(24.0,27.0)	<0.001	N/A	N/A	N/A	31 (PDD)
16	Saul Martínez-Horta,2021, Spain	140	393	Y (CD)	PD-CRS. NOT MOCA	75.1±4	N/A	N/A	N/A	N/A	N/A	N/A	N/A	26 (PDD)
17	M.Petrou, 2016, USA	12	24	Y (CD)	CI if <26	25.3 ± 2.2	0.7	N/A	N/A	N/A	Lower volume – greater CI	611.4 ± 38.3	0.02	N/A
18	J. Park, 2023	Diabetes: 76 Prediabetes: 90 96	N/A	Y (CD)	N/A	DM vs no DM: 0.002 Pre-DM vs no DM: 0.008	N/A	N/A	N/A	N/A	N/A	N/A	N/A	N/A
19	M. Giuntini, 2014	50 50	N/A	Y (CD)	N/A	N/A	N/A	At follow-up: PD-DM: 26.50 ± 3.08 PD-no DM: 27.38 ± 3.2	N/A	N/A	N/A	N/A	N/A	N/A
20	Bosco D., 2012, Italy	53	57	Y(Dementia)	N/A	N/A	N/A	N/A	16.6 ± 3.9 for PD-D and 26.1 ± 1.6 for PD	0.01	N/A	N/A	N/A	53 (PDD)

21	Jolie D. Barter, 2023	34	143	Y (CD)	N/A	24 ± 4.7	N/A	N/A	N/A	N/A	N/A	N/A	N/A	N/A
22	Miyake Y., 2010, Japan	10	368	N/A	N/A	N/A	N/A	N/A	N/A	N/A	N/A	N/A	N/A	N/A
23	Natalia Palacios et al., 2011, USA	Total N=147,096	N/A	N/A	N/A	N/A	N/A	N/A	N/A	N/A	N/A N/A	N/A	N/A	N/A

Note: preclinical studies are not added to this table

Abbreviations: PD – Parkinson's disease; T2DM – Type 2 diabetes mellitus; IR – Insulin resistance; CD – Cognitive decline; PDD – Parkinson's disease dementia; MoCA – Montreal Cognitive Assessment; MMSE – Mini-Mental State Examination; GMV – Gray matter volume; SD – Standard deviation; N/A – Not available, PD-CRS – Parkinson's Disease Cognitive Rating Scale.
